# Update on trabecular bone score

**DOI:** 10.20945/2359-3997000000559

**Published:** 2022-11-10

**Authors:** Telma Palomo, Patricia Muszkat, Fernanda G. Weiler, Patricia Dreyer, Cynthia M. A. Brandão, Barbara C. Silva

**Affiliations:** 1 Fleury Medicina e Saúde Serviço de Densitometria Óssea São Paulo SP Brasil Serviço de Densitometria Óssea, Fleury Medicina e Saúde, São Paulo, SP, Brasil; 2 Unidade de Endocrinologia Santa Casa de Belo Horizonte Belo Horizonte MG Brasil Unidade de Endocrinologia, Santa Casa de Belo Horizonte, Belo Horizonte, MG, Brasil; 3 Unidade de Endocrinologia Hospital Felício Rocho Belo Horizonte MG Brasil Unidade de Endocrinologia, Hospital Felício Rocho, Belo Horizonte, MG, Brasil; 4 Centro Universitário de Belo Horizonte Departamento de Medicina Belo Horizonte MG Brasil Departamento de Medicina, Centro Universitário de Belo Horizonte (UNI-BH), Belo Horizonte, MG, Brasil

**Keywords:** Trabecular bone score, dual-energy X-ray absorptiometry, osteoporosis, fracture risk, secondary osteoporosis

## Abstract

Trabecular bone score (TBS) is an indirect and noninvasive measure of bone quality. A low TBS indicates degraded bone microarchitecture, predicts osteoporotic fracture, and is partially independent of clinical risk factors and bone mineral density (BMD). There is substantial evidence supporting the use of TBS to assess vertebral, hip, and major osteoporotic fracture risk in postmenopausal women, as well as to assess hip and major osteoporotic fracture risk in men aged > 50 years. TBS complements BMD information and can be used to adjust the FRAX (Fracture Risk Assessment) score to improve risk stratification. While TBS should not be used to monitor antiresorptive therapy, it may be potentially useful for monitoring anabolic therapy. There is also a growing body of evidence indicating that TBS is particularly useful as an adjunct to BMD for fracture risk assessment in conditions associated with increased fracture risk, such as type-2 diabetes, chronic corticosteroid excess, and other conditions wherein BMD readings are often misleading. The interference of abdominal soft tissue thickness (STT) on TBS should also be considered when interpreting these findings because image noise can impact TBS evaluation. A new TBS software version based on an algorithm that accounts for STT rather than BMI seems to correct this technical limitation and is under development. In this paper, we review the current state of TBS, its technical aspects, and its evolving role in the assessment and management of several clinical conditions.

## INTRODUCTION

Osteoporosis is a common disease characterized by low bone strength leading to bone fragility and a consequent susceptibility to fractures (1). Bone mineral density (BMD) measurement using dual-energy X-ray absorptiometry (DXA) is the standard tool for the diagnosis of osteoporosis. BMD accounts for 60%-70% of the variation in bone strength (2); additionally, fracture risk increases with decreasing BMD (3). However, most individuals with a fragility fracture are found to have BMD values within the osteopenic or even normal range (4); this indicates that other variables also influence fracture occurrence independent of the BMD. Certain clinical risk factors (CRF), such as older age and personal history of osteoporotic fractures, among others, combined with BMD improves fracture prediction as compared to BMD alone (5).

Additionally, it is important to understand that bone strength is also affected by bone quality, an umbrella term that describes a set of characteristics such as the structural and material properties of the bone, both of which are affected by bone turnover rate. The structural properties of bone include geometry and microarchitecture (trabecular thickness, connectivity, separation and number, and cortical thickness and porosity), whereas the material properties include bone mineral content (crystal size and orientation) and collagen composition, as well as damage accumulation (6).

Investigation of bone microarchitecture and bone remodeling by histomorphometric or micro computed tomography (micro-CT) analysis of the transiliac crest bone biopsy is highly informative (7), but it is an invasive procedure or not widely available. Other noninvasive technologies include high-resolution peripheral quantitative computed tomography (HR-pQCT) and micro-magnetic resonance imaging (micro-MRI); these methods can assess bone microarchitecture, but their use is limited in clinical settings due to high costs when compared to DXA and serve largely as research tools (8,9). Hence, there is a need to develop noninvasive clinically available techniques to evaluate bone quality. To this end, trabecular bone score (TBS), a texture index derived from a lumbar spine (LS) DXA image, is a novel technique that may be used to improve fracture risk prediction beyond that offered by a combination of BMD and CRFs (10).

## TBS – GENERAL PRINCIPLES

**Figure 1:**
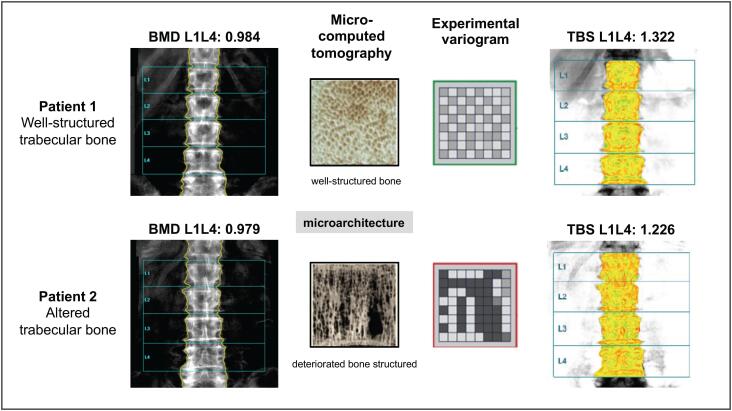
Concept of TBS – the figure presents an example of two different patients with equivalent BMD but different TBS

TBS: Trabecular Bone Score; BMD: Bone Mineral Density

TBS is a textural index that evaluates pixel gray-level variations in lumbar spine DXA image providing an indirect parameter of trabecular architecture. It is determined by constructing a variogram of the projected image (containing the region of interest) and computing the sum of the square of gray-level differences between pixels at a specific distance (Figure 1), followed by calculating the slope of the “log-log transform” of this variogram (11). The TBS software (TBS iNsight; Medimaps Group, Geneva, Switzerland) generates results (unitless) for the whole LS (L1 to L4) and each vertebra using the same region of interest as for BMD. Vertebrae excluded from BMD calculation (fractures or osteoarthritis) may also be excluded from the TBS analysis (10). Because the DXA image is usually retrievable, regardless of the timing of obtaining the image, TBS can be readily applied to any available DXA image obtained from a GE Lunar (Prodigy and iDXA; Madison, WI, USA) or Hologic (Delphi, QDR 4500, and Discovery; Waltham, MA, USA) densitometers (12). A low TBS value is associated with a deteriorated bone architecture; conversely, a high TBS value is correlated with a better bone structure (10). The TBS cutoff points are discussed in a subsequent section.

It is important to note that TBS does not directly assess bone microarchitecture because DXA lacks the resolution to detect bone trabeculae.

TBS was developed using two-dimensional projections of three-dimensional micro-CT images of human cadaveric bone specimens (13). There were significant correlations between TBS and bone volume fraction, trabecular spacing, and the number of trabeculae obtained using cadaveric vertebra, femoral neck, and distal radius samples. Eventually, the technique was extended to DXA images acquired *ex vivo* in cadaveric vertebrae, and significant correlations were found between trabecular indices obtained by micro-CT and TBS; notably, these results were independent of the patient’s BMD (14,15).

However, some studies have failed to demonstrate a significant correlation between TBS and microarchitecture parameters (16,17). *In vivo* studies have reported weak to moderate correlations between TBS and microarchitecture (18–20). Nevertheless, irrespective of the structural properties assessed by TBS, its clinical utility derives from its demonstrated ability to predict fractures.

## FRACTURE RISK ASSESSMENT

In postmenopausal women, several studies (summarized in Table 1) have consistently supported the ability of TBS to predict isolated vertebral or hip, and major osteoporotic fractures (MOF: clinical spine, hip, forearm, and humerus) independently of BMD and CRF (21–29). On average, every 1-point standard deviation (SD) decline in the TBS leads to a 30%-40% increase in the risk of fragility fractures in postmenopausal women.

**Table 1:** A summary of longitudinal studies evaluating trabecular bone score and fracture risk in women

Study	Study population and place of research	Mean age (years)	Mean follow-up time (years)	Outcome (number of fractures)	Adjustments	HR or OR per SD decrease in TBS (95% CI)
Hans and cols., 2011 (21)	29,407 women aged ≥ 50 years (Canada)	65.4	4.7	MOF by Fx codes in health service records (n = 1,668)	Age, LS-BMD, and a combination of clinical risk factors*	HR = 1.17 (1.09-1.25)
				Clinical vertebral Fx (n = 439)		HR = 1.14 (1.03-1.26)
				Hip Fx (n = 293)		HR = 1.47 (1.30-1.67)
Boutroy and cols., 2013 (22)	560 postmenopausal Caucasian women (France)	66.2	7.8	Fragility Fx at any site (except head, toes. and fingers), confirmed by radiographs (n = 94)	Age, weight, and prevalent fracture at baseline	OR = 1.34 (1.04-1.73)
Briot and cols., 2013 (23)	1,007 postmenopausal Caucasian women aged >55 years (Europe)	65.9	6.0	Clinical self-reported osteoporotic Fx, confirmed by radiographs (n = 82)	None	OR = 1.62 (1.30-2.01)
				Vertebral Fx by radiographs (n = 46)		OR = 1.54 (1.17-2.03)
Iki and cols., 2014 (24)	665 women aged ≥ 50 years (Japan)	64.1	8.3	Vertebral Fx by VFA (n = 92)	Age, LS-BMD, and prevalent vertebral deformity	OR = 1.52 (1.16-2.00)
Leslie and cols., 2014 (25)	33,352 women aged 40-100 years (Canada)	63.2	4.7	MOF by Fx codes in health service records (n = 1,872)	Age, time since baseline, clinical risk factors**, and LS-BMD/Age, time since baseline, clinical risk factors**, and femoral neck BMD	HR = 1.17 (1.11-1.23)/HR = 1.18 (1.12-1.23)
				Death (1,754)		HR = 1.26 (1.19-1.32)/HR = 1.20 (1.14–1.26)
McCloskey and cols., 2015 (26)	33,352 women aged 40-100 years (Canada)	63.2	4.7	MOF (excluding hip) by Fx codes in health service records (n = 1,639)	Age, time since baseline, femoral neck BMD, and clinical risk factors***	HR = 1.18 (1.12-1.24)
				Hip Fx (306)		HR = 1.23 (1.09-1.38)
				Death (1,754)		HR = 1.20 (1.14-1.26)
Popp and cols., 2016 (27)	556 postmenopausal elderly women (Switzerland)	76.1	2.7	Clinical fragility Fx (n = 52: 20 forearms, 10 vertebral, 9 humeral, 6 hip, 3 ankle, 2 pelvis, 1 clavicular, and 1 elbow)	Age, BMI, and lowest BMD	HR = 1.87 (1.38-2.54)
McCloskey and cols., 2016 (28)	17,809 individuals (10,507 women); a meta-analysis of 14 international population-based cohorts (North America, Asia, Australia, and Europe)	72	6.1	MOF (n = 1,109)	Age, time since baseline, and FRAX score (with BMD)	HR = 1.31 (1.21-1.42)
				Hip Fx (n = 298)		HR = 1.29 (1.09-1.52)
Su and cols., 2017 (29)	1,950 community-dwelling women aged ≥ 65 years (Hong Kong)	72.5	8.8	MOF (n = 215)	FRAX score (with BMD)	HR = 1.32 (1.13-1.54)
Tamaki and cols., 2019 (35)	1,541 women aged ≥ 40 years (Japan)	58.1	10	MOF by interviews or mail surveys (n = 67)	FRAX score (with BMD)	OR = 1.46 (1.08-1.98)
				Hip Fx (n = 11)	FRAX score (with BMD)	OR = 1.73 (0.82-3.65)
Greendale and cols., 2020 (34)	1,362 premenopausal women (United States)	46.4	22	Any type of Fx (except face, skull, toes, and fingers) detected by self-reported questionnaire – 75% confirmed by medical reports (n = 292, 111 minimal trauma)	Age, race/ethnicity, BMI, bone active medications, LS BMD	HR = 0.95 (0.79-1.14)
	891 early postmenopausal women (United States)	54.1		Any type of Fx (n = 141, 60 minimal trauma)		HR = 1.03 (0.79-1.33)

BMD: bone mineral density; BMI: body mass index; FRAX: The WHO Fracture Risk Assessment tool; Fx: fracture; HR: hazard ratio; LS: lumbar spine; MOF: major osteoporotic fracture (hip, clinical spine, forearm, and humerus); OR: odds ratio; TBS: trabecular bone score.

* Clinical risk factors: ambulatory diagnostic groups comorbidity score, rheumatoid arthritis, chronic obstructive pulmonary disease, diabetes, substance abuse, body mass index, prior osteoporotic fracture, systemic corticosteroid use in the last year, and osteoporosis treatment in the last year.

** Clinical risk factors: secondary osteoporosis, rheumatoid arthritis, chronic obstructive pulmonary disease (smoking proxy), high alcohol use, body mass index, previous fracture, and glucocorticoid use > 90 days.

*** Clinical risk factors: secondary osteoporosis, rheumatoid arthritis, smoking, alcohol use, body mass index, previous fracture, and glucocorticoids.

The relationship between TBS and the incidence of fractures has also been assessed in men over the age of 50 years. Most of the longitudinal publications have reported that TBS can independently predict MOF and hip fractures in this population (28–32) (Table 2). Only a few reports have provided information about vertebral fractures in older men, with mixed results (30,33).

**Table 2:** A summary of longitudinal studies evaluating trabecular bone score and fracture risk in men

Study	Study population and place of research	Mean age (years)	Mean follow-up (years)	Outcome (number of subjects)	Adjustments	HR or OR per SD decrease in TBS (95% CI)
Leslie and cols., 2014 (30)	3,620 men aged ≥ 50 years (Canada)	67.6	4.5	MOF by Fx codes in health service records (n = 183)	Clinical FRAX score, osteoporosis treatment, and LS-BMD	HR = 1.08 (0.92-1.26)
				Clinical vertebral Fx (n = 91)		HR = 1.02 (0.81-1.27)
				Hip Fx (n = 46)		HR = 1.44 (1.07-1.94)
Iki and cols., 2015 (31)	1,805 community-dwelling men aged ≥ 65 years (Japan)	73	4.5 (median)	MOF by interviews or mail and telephone surveys (n = 22)	FRAX score (with BMD)	OR = 1.76 (1.16-2.67)
McCloskey and cols., 2016 (28)	17,809 individuals (7,302 men); a meta-analysis of 14 international population-based cohorts (North America, Asia, Australia, and Europe)	72	6.1	MOF (n = 1,109)	Age, time since baseline, and FRAX score (with BMD)	HR = 1.35 (1.21-1.49)
				Hip Fx (n = 298)		HR = 1.27 (1.06-1.53)
Schousboe and cols., 2016 (32)	5,863 community-dwelling men aged ≥ 65 years (United States)	73.7	10	MOF by mail surveys and confirmed by radiographs (n = 448)	FRAX score (with BMD) and prevalent radiographic vertebral Fx	HR = 1.27 (1.17-1.39)
				Hip Fx (n = 181)		HR = 1.20 (1.05-1.39)
Schousboe and cols., 2017 (33)	5,831 community-dwelling men aged ≥ 65 years (United States)	73.7	11.5	Clinical vertebral Fx (n = 202)	Age and LS-BMD	OR = 1.19 (1.02-1.38)
	4,309 community-dwelling men aged ≥ 65 years	Not described	4.6	Radiographic vertebral Fx (n = 196)		OR = 1.11 (0.94-1.30)
Su and cols., 2017 (29)	1,923 community-dwelling men aged ≥ 65 years (Hong Kong)	72.3	9.9	MOF (n = 126)	FRAX score (with BMD)	HR = 1.38 (1.15-1.65)

BMD: bone mineral density; FRAX: The WHO Fracture Risk Assessment tool; Fx: fracture; HR: hazard ratio; LS: lumbar spine; MOF: major osteoporotic fracture (hip, clinical spine, forearm, and humerus); OR: odds ratio; TBS: trabecular bone score.

Recently, Greendale and cols. (34) evaluated TBS values and fracture incidence in pre and early postmenopausal women. Minimal trauma but also traumatic fractures were considered in this study (Table 1). For premenopausal women (n = 1,362; mean age = 46.4 years), TBS was related to fracture risk in models adjusted for age, body mass index (BMI), race/ethnicity, and bone active medications. However, TBS was no longer a predictor of fracture incidence after adjusting for LS or femoral neck (FN) BMD. On the other hand, in early postmenopausal women (n = 891; mean age = 54.1 years), TBS was not associated with fracture risk even in models without BMD adjustment, but the power to detect this association was insufficient.

To summarize, so far, the existing literature supports the use of TBS to assess the risk of MOF, vertebral, and hip fractures in postmenopausal women, as well as that of MOF and hip fractures in men over the age of 50 years.

## TBS and FRAX (THE WORLD HEALTH ORGANIZATION (WHO) FRACTURE RISK ASSESSMENT TOOL)

The FRAX tool is an online fracture risk assessment algorithm developed by the Collaborating Center for Metabolic Bone Diseases of the World Health Organization in 2008 to assess the 10-year fracture probability in people aged 40 to 90 years. FRAX contains country-specific prediction models based on underlying fracture risk and mortality for the reference population. Its calculation is based on the most frequently studied CRFs namely age, BMI, history of prior fractures, parental hip fractures, current smoking status, chronic use of glucocorticoids (GC), rheumatoid arthritis, secondary osteoporosis, and alcohol intake; the use of FN-BMD is not mandatory. The algorithm provides the 10-year probability of an individual sustaining a MOF and hip fractures (FN, intertrochanteric, or subtrochanteric) (36).

Several cross-sectional and prospective studies including different populations have consistently shown that TBS may improve FRAX accuracy in postmenopausal women and older men (25,26,28,31,32,37). Data from the Manitoba Study (26), large population-based research comprising > 33,000 people aged 40-99 years followed up over an average of 4.7 years, have been used to derive potential correction factors for calculating a TBS-adjusted FRAX score. In the Manitoba cohort, even after fully adjusting for FRAX risk variables, TBS was found to be an independent predictor of MOF (excluding hip fracture) (hazard ratio/standard deviation (HR/SD): 1.18, 95% confidence intervals, CI: 1.12-1.24), hip fracture (HR/SD: 1.23, 95% CI: 1.09-1.38), and mortality (HR/SD: 1.20, 95% CI 1.14-1.26) (Table 1).

McCloskey and cols. (28) conducted a wide meta-analysis including data from 14 prospective population-based cohorts (using individual-level data from 17,809 men and women) from North America, Asia, Australia, and Europe. They observed that TBS was independently associated with the risk of MOF, and mainly hip fractures at all ages, in both sexes (Tables 1 and 2). Moreover, the incorporation of a TBS adjustment factor allowed for slightly greater risk stratification for both MOF and hip fractures. In short, the combination of TBS with CRFs and BMD enhanced the performance of FRAX to predict MOF and hip fractures compared with using either TBS or FRAX risk variables alone. Similar results have been reported in studies including older Chinese men and Japanese women (29,35). In contrast, Holloway and cols. (38), in a longitudinal study involving 591 Australian men aged 40-90 years, concluded that fracture prediction using FRAX was not substantially improved by TBS adjustment. Furthermore, Martineau and cols. have shown that the clinical impact of TBS-adjusted FRAX was greater in individuals close to the FRAX-based therapeutic intervention threshold, whereas the TBS adjustment was unlikely to reclassify someone with a very low unadjusted FRAX score into a high-risk (39). Also, it was reported that the utility of TBS-adjusted FRAX was greatest in postmenopausal women under 65 years old, owing to the significant interaction between TBS and age.

Finally, a small study suggested that the adjustment of FRAX by TBS may be useful when there is a discordance between LS and FN BMD, particularly in patients with no history of osteoporotic fractures (40).

## APPLICATION OF TBS IN GUIDING CLINICAL DECISIONS

Recommendations from the International Society of Clinical Densitometry (ISCD) and the European Society for Clinical and Economic Aspects of Osteoporosis – Osteoarthritis, and Musculoskeletal Diseases (ESCEO) support the use of TBS to assess fracture risk in postmenopausal women and men over the age of 50 years (37,41). Additionally, TBS was proposed to be evaluated in postmenopausal women with type 2 diabetes (T2D) to predict MOF (42).

However, there is no consensus about TBS cutoff points. Cormier proposed that TBS values ≥ 1.350 should be considered normal, while a TBS = 1.200-1.350 is consistent with “partially degraded” bone, and TBS ≤ 1.200 indicates “degraded” bone in postmenopausal women (43). In this regard, McCloskey and cols. (28) published an interesting study that related TBS thresholds with fracture risk (low, intermediary, and high-risk groups). The two threshold values are 1.230 and 1.310, with no differences between the sexes. Individuals in the lower two tertiles, i.e., with high (TBS < 1.230) or intermediate risk (TBS = 1.230-1.310), present the highest risk for MOF compared with the lowest risk tertile (TBS > 1.310). For the Latin American population, the manufacturer proposed different TBS values (published as abstract (44) for women and unpublished data for men) for both men and women that are higher than those proposed by McCloskey and Cormier. For men, the thresholds are 1.258 and 1.338, while for women 1.267 and 1.347. TBS tertiles reported for Brazilian elderly women are similar to those proposed for Latin American women (45).

In 2022, Kalkwarf and cols. (46) presented a robust reference range for the pediatric population using the new TBS software (pre-release version 4.0) and determined its predictive value for bone fragility in childhood and adolescence.

While a low TBS is associated with a greater risk of fracture, a threshold value to initiate treatment has not been determined yet; therefore, the ISCD has recommended against the use of TBS as a single parameter to guide treatment decisions (37). The ISCD suggestion was to adjust FRAX probabilities using TBS values to assist in treatment decisions (37).

Another important issue regarding TBS is its reproducibility, which is critical for its use in disease progression and therapeutic monitoring. This can be ensured by precision assessment and calculation of the least significant change (LSC), which determines when a difference in the measurement is statistically significant or within the range of error of the test. ISCD recommends a conservative estimate of 5.8% for TBS-LSC as the threshold to consider the use of TBS to monitor changes in individual patients on osteoporosis medications unless the individual site performed their own health service LSC (47).

Approved bisphosphonates, whether administered orally or parenterally, reduce bone remodeling, increase BMD, and reduce fracture risk, but are not known to alter the bone structure. There is a very good consistency across the studies that patients treated with oral bisphosphonates (alendronate, risedronate, and ibandronate) or zoledronic acid for two or more years show minimal, non-significant changes in TBS (37,48–50). Therefore, TBS is not an appropriate measure to follow-up on the progress in patients on bisphosphonates; likewise, the ISCD does not recommend TBS to monitor bisphosphonate therapy (47). Although a study described that the increase in the TBS with denosumab was greater than that obtained with bisphosphonates, the results were insufficient to support the use of TBS for routine monitoring of these patients (51). In general, the literature suggests that TBS should not be used for monitoring patients taking antiresorptive drugs for periods up to three years (47).

Nevertheless, TBS can be potentially useful for monitoring anabolic therapy. Anabolic drugs (teriparatide and abaloparatide) can increase the mean TBS when used over 2-3 years. However, the response may not be uniform; the rate of change beyond the LSC is estimated to be about 52% over 24 weeks for abaloparatide and probably less for teriparatide (50,52,53). Although a change in TBS alone is not recommended to ascertain a good response to an anabolic agent, TBS monitoring in these patients may offer additional information beyond BMD and bone turnover markers (47). Data regarding the potency of romosozumab based on the TBS is lacking. In a recent small study, 10 patients treated with romosozumab for six months had a modest increase in TBS (2.53%; p = 0.04), that did not reach the LSC (5.8%) (54).

## TBS AND FRACTURE RISK PREDICTION IN PATIENTS WITH DIABETES AND SECONDARY OSTEOPOROSIS

**Figure 2:**
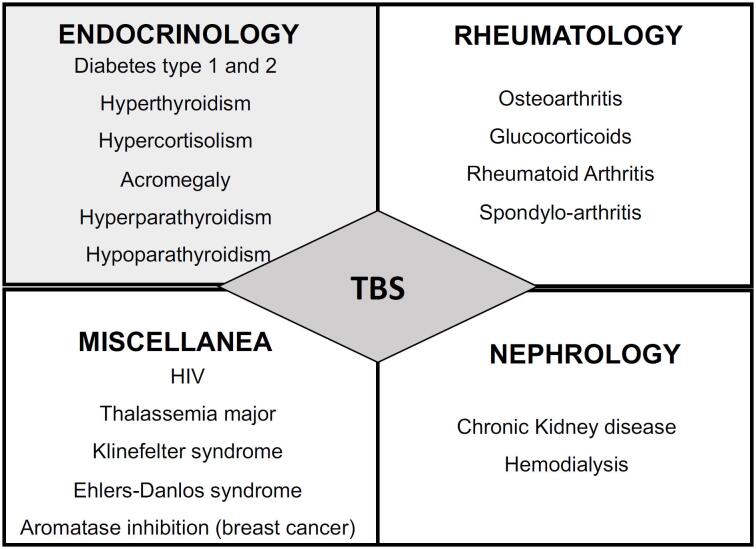
A list of special conditions (causing secondary osteoporosis) known to increase fracture risk in which TBS has been demonstrated to add some clinical value

A growing number of studies have assessed TBS in various conditions known to increase the risk of fragility fractures (10) and many of them have corroborated the ability of TBS to predict such fractures in patients with secondary osteoporosis (55,56). Figure 2 presents a list of some of these pathologies.

TBS: Trabecular Bone Score.

In general, compared to control subjects, TBS was reported to be lower in patients with diabetes (42,57–59), primary hyperparathyroidism (60–64), acromegaly (65–68), anorexia nervosa (69,70), hypercortisolism (71–73), primary aldosteronism (especially in women) (74), with prolonged GC exposure (75–84), rheumatoid arthritis and rheumatic disease (85–87), aromatase inhibition (88), kidney transplant recipients (89), as well as in patients on hemodialysis (90,91). In differentiated thyroid carcinomas, TBS was found to be lower in patients receiving long-term suppressive doses of thyroid-stimulating hormone than in patients receiving shorter-term therapy (92–94). Some other studies also evaluated TBS in thalassemia major (95,96), Human Immunodeficiency virus-acquired immunodeficiency syndrome (HIV-AIDS) (97,98), Klinefelter syndrome (99), Ehlers-Danlos syndrome (100), Down syndrome (101), and short stature (102).

In this section, we delve deeper into the role of TBS in the assessment of bone health in patients with diabetes mellitus and those on long-term GC exposure.

## DIABETES

Previous studies have robustly shown the association between diabetes and bone fragility, and it is well-known that the skeleton is affected by both type 1 and 2 diabetes mellitus, leading to an increased risk of fractures (56,103). Interestingly, there is a paradoxical relationship between type 2 diabetes (T2D), BMD, and fractures (61). Compared to the general population, patients with T2D are at an increased risk for fragility fractures at all skeletal sites (104–106) despite having comparable or even higher BMD values measured by DXA (107,108). In other words, BMD may underestimate fracture risk in T2D (109). Alterations in skeletal properties or bone quality are possible explanations for this T2D-related skeletal fragility (110), and TBS could be useful for fracture risk assessment in these patients (59).

In 2013, Leslie and cols. (42) were the first to examine the association between TBS and the incidence of fractures in 29,407 women above the age of 50 years from the province of Manitoba, Canada, including 2,356 with diabetes (mostly T2D). Interestingly, compared to controls, women with diabetes had higher baseline BMD at all sites, but lower TBS, even after adjusting for multiple confounding variables. Furthermore, over a mean of 4.7 years of follow-up, the incidence of MOF was greater in women with diabetes (7.4%, n = 175) than in non-diabetics (5.5%, n = 1,493; p < 0.001). They reported that TBS predicted MOF independently of BMD in women with diabetes (HR: 1.27, 95% CI: 1.10-1.46), similar to those without diabetes (HR: 1.31, 95% CI: 1.24-1.38). Other studies have also confirmed that despite greater BMD values, those with T2D have lower TBS than controls (55,58). Another study found a greater prevalence of morphometric vertebral fractures in postmenopausal women with T2D (34.3%) than in controls (18.7%, p = 0.01) (111). Vertebral fractures were associated with lower values of TBS (area under the curve, AUC: 0.69; p < 0.0001) and FN-BMD (AUC: 0.63; p < 0.004).

A recent meta-analysis of 40,508 individuals (35,546 women and 4962 men; 4,269 patients with diabetes) showed that, overall, T2D was associated with decreased TBS (more pronounced in women) (61). However, there was evidence of substantial heterogeneity among studies – most of them used unadjusted TBS values, and only a few adjusted for parameters that may directly affect TBS, such as age, BMI, LS-BMD, and the TBS software (56,61).

In summary, the relationship between T2D and TBS is mixed. Several studies (42,57,112–117), but not all (58,111), have shown that TBS is lower in patients with diabetes, especially in those with poor glycemic control, disease complications, and/or longer duration of disease. This discrepancy could also be attributed to the differences in sample size, duration of diabetes, HbA1c levels, and multifactorial pathophysiology of bone fragility in diabetes, which demands further investigation (45,57,59,61). Recently, our research group reported that the effect of abdominal soft tissue thickness (STT) should be considered when interpreting TBS in patients with T2D in whom increased abdominal adiposity may artifactually reduce TBS values (45).

Another study examined TBS in 119 patients with type 1 diabetes (T1D) (59 males, 60 premenopausal females; mean age = 43.4 years) and 68 matched healthy controls and found that TBS was comparable in T1D patients and non-diabetic controls, but was lower in T1D patients with existing clinical fractures (n = 24) than in controls (118). Using a multivariate model, TBS (p = 0.049) and HbA1c (p = 0.036) were found to be independently associated with prevalent fractures in T1D patients. A few other studies have examined the differences in TBS between T1D patients and healthy controls and have reported heterogeneous results (119–121).

## LONG-TERM GC EXPOSURE

It is well known that prolonged GC exposure is associated with increased fracture risk and a significant age-adjusted decrease in TBS, but not in LS-BMD (75,76). Two independent studies have shown that TBS differentiated subjects according to chronic GC exposure. Paggiosi and cols. studied 484 women (aged 55-79 years), allocated into 3 groups: 64 taking prednisolone ≥ 5 mg/day for > 3 months, 141 who had sustained a recent MOF, and 279 healthy women (75). Compared to healthy women, those with a recent fracture had lower age-adjusted LS-BMD and TBS Z-scores. In contrast, women on GC had comparable age-adjusted LS BMD but lower adjusted TBS Z-scores (p < 0.001) than healthy controls. TBS (AUC = 0.721), but not LS BMD (AUC = 0.572), was able to discriminate between GC-treated and GC-NAÏVE women.

Leib and Winzenrieth also assessed TBS, BMD, and osteoporotic fractures in 416 individuals (mean age = 63.4 years; 72 males) taking prednisone ≥ 5 mg/day for ≥ 3 months and compared them to 1,104 sex-, age-, and BMI-matched controls (76). Prevalent osteoporotic fractures were present in 16.3% of cases and 13.1% of GC-naïve subjects (p = 0.12). Also, TBS and BMD Z-scores at the hip sites, but not LS-BMD, were lower in the GC group. In the GC-naïve subjects, both TBS and LS-BMD were able to differentiate between patients with and without fractures. In contrast, in the GC group, TBS (but not LS-BMD) was able to distinguish between fractured and non-fractured individuals. Using a multivariate model, the authors showed that each point decrease in SD of TBS conferred a 51% greater risk of prevalent fracture (95% CI: 1.23-1.86).

In 2019, Florez and cols. (79) published a study including 127 subjects (mean age = 62 years; 63% females) treated with GC for different autoimmune diseases that led to osteoporosis over a mean period of 47.7 months. About 28% of these patients had some type of fracture and 17% of them had prevalent vertebral fractures. While the BMD T-score was used to establish the diagnosis of osteoporosis in 29% of cases, 52% of the study population had degraded microarchitecture as measured by TBS. Another study evaluated 627 patients with asthma being treated with GC and an equal number of non-asthmatic controls (122). TBS values were lower in those with asthma compared to control subjects (1.320 versus 1.360, respectively; p = 0.001), whereas LS-BMD was similar between the two groups.

## IMPACT OF HETEROTOPIC OSSIFICATION AND ABDOMINAL STT ON TBS

There is evidence that TBS is less affected by the presence of heterotopic ossifications that may typically overestimate LS-BMD. Data from some studies suggest that neither osteoarthritic changes in elderly women nor lumbar syndesmophytes in men with spondyloarthritis influenced TBS results (123–125). The presence of a vertebral fracture also seems to have less impact on TBS, whereas falsely elevate measured BMD (126). In contrast, White and cols. found that vertebral exclusion, as per ISCD recommendations, generally, tends to lower TBS (but not always) and may result in relevant changes in calculated fracture risk using FRAX (127). Until more data is available, vertebrae excluded from BMD calculation (fractures or osteoarthritis) should also be excluded from the TBS analysis (10).

In contrast, similar to what occurs in BMD measurement (128), TBS analysis may be affected by the amount of local soft tissue, which leads to X-ray attenuation, degrading the image texture. Excess abdominal soft tissue may artificially reduce TBS values due to deleterious effects of image noise (129), particularly in subjects with T2D in whom increased abdominal adiposity may artifactually reduce TBS values.

To address this issue, the current TBS algorithm adjusts for BMI as a surrogate marker for regional STT (10). The manufacturer recommends that TBS only be performed in patients with a BMI of 15-37 kg/m^2^; TBS has not been validated in patients with BMIs outside of this range. However, even with a BMI of 15-37 kg/m^2^, the use of BMI to adjust the TBS calculation is not perfect because it cannot distinguish patients with higher central adiposity from those with a more peripheral fat accumulation (130). Hence, BMI adjustment only indirectly addresses soft tissue interference on the lumbar spine scan area used for TBS analysis, while measuring local STT accounts for this issue directly (131). Recently, our study group showed that in women with T2D, glucose intolerance, and normal glucose metabolism, higher HbA1c levels were associated with greater BMD, higher abdominal STT, and lower TBS values (45). However, after adjusting for local adiposity, TBS differences among groups disappeared, except in the subgroup of women with higher HbA1c levels and longer disease duration. These results indicate that the effect of abdominal STT should be considered when interpreting TBS results obtained from the current commercially available TBS software version, particularly in patients with T2D. A new TBS software version is under development which is based on an algorithm that takes into account STT rather than BMI, seems to correct this technical limitation (131).

In conclusion, this review elaborates on the potential applicability of TBS in clinical practice. This textural index is readily available from spine DXA images and is associated with vertebral and non-vertebral fractures in postmenopausal women and older men. TBS appears to reflect qualitative aspects of skeletal structures that are partially independent of CRFs and DXA BMD measurement, being included as a risk factor in the FRAX tool. However, it should not be used alone to guide clinical decisions, and some limitations, such as the lack of a well-established cutoff point and image noise interference, must be acknowledged. Finally, TBS seems to have a role in anabolic drug response but may not be useful for monitoring antiresorptive therapy.

## References

[1] Klibanski A, Adams-Campbell L, Bassford T, Blair SN, Boden SD, Dickersin K (2001). Osteoporosis prevention, diagnosis, and therapy. JAMA.

[2] Johnell O, Kanis JA, Oden A, Johansson H, De Laet C, Delmas P (2005). Predictive value of BMD for hip and other fractures. J Bone Miner Res.

[3] Marshall D, Johnell O, Wedel H (1996). Meta-analysis of how well measures of bone mineral density predict occurrence of osteoporotic fractures. Br Med J.

[4] Pasco JA, Seeman E, Henry MJ, Merriman EN, Nicholson GC, Kotowicz MA (2006). The population burden of fractures originates in women with osteopenia, not osteoporosis. Osteoporos Int.

[5] Kanis JA, Oden A, Johnell O, Johansson H, De Laet C, Brown J (2007). The use of clinical risk factors enhances the performance of BMD in the prediction of hip and osteoporotic fractures in men and women. Osteoporos Int.

[6] Felsenberg D, Boonen S (2005). The bone quality framework: Determinants of bone strength and their interrelationships, and implications for osteoporosis management. Clin Ther.

[7] Kulak CAM, Dempster DW (2010). Bone histomorphometry: A concise review for endocrinologists and clinicians. Arq Bras Endocrinol Metabol.

[8] Whittier DE, Boyd SK, Burghardt AJ, Paccou J, Ghasem-Zadeh A, Chapurlat R (2020). Guidelines for the assessment of bone density and microarchitecture in vivo using high-resolution peripheral quantitative computed tomography. Osteoporos Int.

[9] Sollmann N, Löffler MT, Kronthaler S, Böhm C, Dieckmeyer M, Ruschke S (2021). MRI-based quantitative osteoporosis imaging at the spine and femur. J Magn Reson Imaging.

[10] Silva BC, Leslie WD, Resch H, Lamy O, Lesnyak O, Binkley N (2014). Trabecular bone score: A noninvasive analytical method based upon the DXA image. J Bone Miner Res.

[11] Silva BC, Bilezikian JP (2014). Trabecular bone score: perspectives of an imaging technology coming of age. Arq Bras Endocrinol Metabol.

[12] Bousson V, Bergot C, Sutter B, Levitz P, Cortet B, Dargent-Molina P (2012). Trabecular bone score (TBS): Available knowledge, clinical relevance, and future prospects. Osteoporos Int.

[13] Pothuaud L, Carceller P, Hans D (2008). Correlations between grey-level variations in 2D projection images (TBS) and 3D microarchitecture: Applications in the study of human trabecular bone microarchitecture. Bone.

[14] Hans D, Barthe N, Boutroy S, Pothuaud L, Winzenrieth R, Krieg MA (2011). Correlations between trabecular bone score, measured using anteroposterior dual-energy X-ray absorptiometry acquisition, and 3-dimensional parameters of bone microarchitecture: An experimental study on human cadaver vertebrae. J Clin Densitom.

[15] Roux JP, Wegrzyn J, Boutroy S, Bouxsein ML, Hans D, Chapurlat R (2013). The predictive value of trabecular bone score (TBS) on whole lumbar vertebrae mechanics: An ex vivo study. Osteoporos Int.

[16] Maquer G, Musy SN, Wandel J, Gross T, Zysset PK (2015). Bone volume fraction and fabric anisotropy are better determinants of trabecular bone stiffness than other morphological variables. J Bone Miner Res.

[17] Maquer G, Lu Y, Dall’Ara E, Chevalier Y, Krause M, Yang L (2016). The initial slope of the variogram, foundation of the Trabecular Bone Score, Is not or is poorly associated with vertebral strength. J Bone Miner Res.

[18] Silva BC, Walker MD, Abraham A, Boutroy S, Zhang C, McMahon DJ (2013). Trabecular bone score is associated with volumetric bone density and microarchitecture as assessed by central QCT and HRpQCT in Chinese-American and white women. J Clin Densitom.

[19] Popp AW, Buffat H, Eberli U, Lippuner K, Ernst M, Richards RG (2014). Microstructural parameters of bone evaluated using HR-pQCT correlate with the DXA-derived cortical index and the trabecular bone score in a cohort of randomly selected premenopausal women. PLoS One.

[20] Amstrup AK, Jakobsen NFB, Moser E, Sikjaer T, Mosekilde L, Rejnmark L (2016). Association between bone indices assessed by DXA, HR-pQCT and QCT scans in post-menopausal women. J Bone Miner Res.

[21] Hans D, Goertzen AL, Krieg MA, Leslie WD (2011). Bone microarchitecture assessed by TBS predicts osteoporotic fractures independent of bone density: The Manitoba study. J Bone Miner Res.

[22] Boutroy S, Hans D, Sornay-Rendu E, Vilayphiou N, Winzenrieth R, Chapurlat R (2013). Trabecular bone score improves fracture risk prediction in non-osteoporotic women: The OFELY study. Osteoporos Int.

[23] Briot K, Paternotte S, Kolta S, Eastell R, Reid DM, Felsenberg D (2013). Added value of trabecular bone score to bone mineral density for prediction of osteoporotic fractures in postmenopausal women: The OPUS study. Bone.

[24] Iki M, Tamaki J, Kadowaki E, Sato Y, Dongmei N, Winzenrieth R (2014). Trabecular bone score (TBS) predicts vertebral fractures in Japanese women over 10 years independently of bone density and prevalent vertebral deformity: The Japanese population-based osteoporosis (JPOS) Cohort study. J Bone Miner Res.

[25] Leslie WD, Johansson H, Kanis JA, Lamy O, Oden A, McCloskey EV (2014). Lumbar spine texture enhances 10-year fracture probability assessment. Osteoporos Int.

[26] McCloskey EV, Odén A, Harvey NC, Leslie WD, Hans D, Johansson H (2015). Adjusting fracture probability by trabecular bone score. Calcif Tissue Int.

[27] Popp AW, Meer S, Krieg MA, Perrelet R, Hans D, Lippuner K (2016). Bone mineral density (BMD) and vertebral trabecular bone score (TBS) for the identification of elderly women at high risk for fracture: The SEMOF cohort study. Eur Spine J.

[28] McCloskey EV, Odén A, Harvey NC, Leslie WD, Hans D, Johansson H (2016). A meta-analysis of trabecular bone score in fracture risk prediction and its relationship to FRAX. J Bone Miner Res.

[29] Su Y, Leung J, Hans D, Lamy O, Kwok T (2017). The added value of trabecular bone score to FRAX® to predict major osteoporotic fractures for clinical use in Chinese older people: The Mr. OS and Ms. OS cohort study in Hong Kong. Osteoporos Int.

[30] Leslie WD, Aubry-Rozier B, Lix LM, Morin SN, Majumdar SR, Hans D (2014). Spine bone texture assessed by trabecular bone score (TBS) predicts osteoporotic fractures in men: The Manitoba Bone Density Program. Bone.

[31] Iki M, Fujita Y, Tamaki J, Kouda K, Yura A, Sato Y (2015). Trabecular bone score may improve FRAX^®^ prediction accuracy for major osteoporotic fractures in elderly Japanese men: The Fujiwara-kyo osteoporosis risk in men (FORMEN) Cohort Study. Osteoporos Int.

[32] Schousboe JT, Vo T, Taylor BC, Cawthon PM, Schwartz AV, Bauer DC (2016). Prediction of incident major osteoporotic and hip fractures by trabecular bone score (TBS) and prevalent radiographic vertebral fracture in older men. J Bone Miner Res.

[33] Schousboe JT, Vo TN, Langsetmo L, Taylor BC, Cawthon PM, Schwartz AV (2017). Association of trabecular bone score (TBS) with incident clinical and radiographic vertebral fractures adjusted for lumbar spine BMD in older men: A prospective cohort study. J Bone Miner Res.

[34] Greendale GA, Huang MH, Cauley JA, Harlow S, Finkelstein JS, Karlamangla AS (2020). Premenopausal and early postmenopausal trabecular bone score (TBS) and fracture risk: Study of Women’s Health across the Nation (SWAN). Bone.

[35] Tamaki J, Iki M, Sato Y, Winzenrieth R, Kajita E, Kagamimori S (2019). Does Trabecular Bone Score (TBS) improve the predictive ability of FRAX^®^ for major osteoporotic fractures according to the Japanese population-based osteoporosis (JPOS) cohort study?. J Bone Miner Res.

[36] University of Sheffield UK FRAX® WHO fracture risk assessment tool.

[37] Silva BC, Broy SB, Boutroy S, Schousboe JT, Shepherd JA, Leslie WD (2015). Fracture risk prediction by non-BMD DXA measures: The 2015 ISCD official positions Part 2: Trabecular bone score. J Clin Densitom.

[38] Holloway KL, Mohebbi M, Betson AG, Hans D, Hyde NK, Brennan-Olsen SL (2018). Prediction of major osteoporotic and hip fractures in Australian men using FRAX scores adjusted with trabecular bone score. Osteoporos Int.

[39] Martineau P, Leslie WD, Johansson H, Oden A, McCloskey EV, Hans D (2017). Clinical utility of using lumbar spine trabecular bone score to adjust fracture probability: The Manitoba BMD cohort. J Bone Miner Res.

[40] Goh TS, Kim E, Jeon YK, Hwangbo L, Kim IJ, Pak K (2021). Spine-hip discordance and FRAX assessment fracture risk in postmenopausal women with osteopenia from concordant diagnosis between lumbar spine and femoral neck. J Clin Densitom.

[41] Harvey NC, Glüer CC, Binkley N, McCloskey EV, Brandi ML, Cooper C (2015). Trabecular bone score (TBS) as a new complementary approach for osteoporosis evaluation in clinical practice. Bone.

[42] Leslie WD, Aubry-Rozier B, Lamy O, Hans D (2013). TBS (trabecular bone score) and diabetes-related fracture risk. J Clin Endocrinol Metab.

[43] Cormier C, Lamy O, Poriau S (2012). TBS in routine clinical practice: Proposals of use. Medimaps.

[44] Camargos BM, Elizondro Alan LJ, Albergaria BH, Clark P (2014). Normative spine TBS data for Latin American women.

[45] Palomo T, Dreyer P, Muszkat P, Weiler FG, Bonansea TCP, Domingues FC (2022). Effect of soft tissue noise on trabecular bone score in postmenopausal women with diabetes: A cross sectional study. Bone.

[46] Kalkwarf HJ, Shepherd JA, Hans D, Gonzalez Rodriguez E, Kindler JM, Lappe JM (2022). Trabecular bone score reference values for children and adolescents according to age, sex, and ancestry. J Bone Miner Res.

[47] Krohn K, Schwartz EN, Chung YS, Lewiecki EM (2019). Dual-energy X-ray absorptiometry monitoring with trabecular bone score: 2019 ISCD official position. J Clin Densitom.

[48] Popp AW, Guler S, Lamy O, Senn C, Buffat H, Perrelet R (2013). Effects of zoledronate versus placebo on spine bone mineral density and microarchitecture assessed by the trabecular bone score in postmenopausal women with osteoporosis: A three-year study. J Bone Miner Res.

[49] Krieg MA, Aubry-Rozier B, Hans D, Leslie WD, Manitoba Bone Density Program (2013). Effects of anti-resorptive agents on trabecular bone score (TBS) in older women. Osteoporos Int.

[50] Senn C, Günther B, Popp AW, Perrelet R, Hans D, Lippuner K (2014). Comparative effects of teriparatide and ibandronate on spine bone mineral density (BMD) and microarchitecture (TBS) in postmenopausal women with osteoporosis: A 2-year open-label study. Osteoporos Int.

[51] McClung MR, Lippuner K, Brandi ML, Zanchetta JR, Bone HG, Chapurlat R (2017). Effect of denosumab on trabecular bone score in postmenopausal women with osteoporosis. Osteoporos Int.

[52] Bilezikian JP, Hattersley G, Fitzpatrick LA, Harris AG, Shevroja E, Banks K (2018). Abaloparatide-SC improves trabecular microarchitecture as assessed by trabecular bone score (TBS): A 24-week randomized clinical trial. Osteoporos Int.

[53] di Gregorio S, del Rio L, Rodriguez-Tolra J, Bonel E, García M, Winzenrieth R (2015). Comparison between different bone treatments on areal bone mineral density (aBMD) and bone microarchitectural texture as assessed by the trabecular bone score (TBS). Bone.

[54] Jeong C, Kim J, Lim Y, Ha J, Kang MI, Baek KH (2021). Effect of Romosozumab on Trabecular bone score compared to anti-resorptive agents in postmenopausal women with osteoporosis. J Bone Metab.

[55] Ulivieri FM, Silva BC, Sardanelli F, Hans D, Bilezikian JP, Caudarella R (2014). Utility of the trabecular bone score (TBS) in secondary osteoporosis. Endocrine.

[56] Shevroja E, Cafarelli FP, Guglielmi G, Hans D (2021). DXA parameters, Trabecular bone score (TBS) and bone mineral density (BMD), in fracture risk prediction in endocrine-mediated secondary osteoporosis. Endocrine.

[57] Dhaliwal R, Cibula D, Ghosh C, Weinstock RS, Moses AM (2014). Bone quality assessment in type 2 diabetes mellitus. Osteoporos Int.

[58] Kim JH, Choi HJ, Ku EJ, Kim KM, Kim SW, Cho NH (2015). Trabecular bone score as an indicator for skeletal deterioration in diabetes. J Clin Endocrinol Metab.

[59] Ho-Pham LT, Nguyen TV (2019). Association between trabecular bone score and type 2 diabetes: A quantitative update of evidence. Osteoporos Int.

[60] Eller-Vainicher C, Filopanti M, Palmieri S, Ulivieri FM, Morelli V, Zhukouskaya V (2013). Bone quality, as measured by trabecular bone score, in patients with primary hyperparathyroidism. Eur J Endocrinol.

[61] Romagnoli E, Cipriani C, Nofroni I, Castro C, Angelozzi M, Scarpiello A (2013). “Trabecular Bone Score” (TBS): An indirect measure of bone micro-architecture in postmenopausal patients with primary hyperparathyroidism. Bone.

[62] dos Santos LM, Ohe MN, Pallone SG, Nacaguma IO, Kunii IS, da Silva REC (2021). Trabecular bone score (TBS) in primary hyperparathyroidism (PHPT): A useful tool?. J Clin Densitom.

[63] Naciu AM, Tabacco G, Falcone S, Incognito GG, Chiodini I, Maggi D (2021). Bone quality as measured by trabecular bone score in normocalcemic primary hyperparathyroidism. Endocr Pract.

[64] Muñoz-Torres M, Manzanares Córdova R, García-Martín A, Avilés-Pérez MD, Nieto Serrano R, Andújar-Vera F (2019). Usefulness of trabecular bone score (TBS) to identify bone fragility in patients with primary hyperparathyroidism. J Clin Densitom.

[65] Hong AR, Kim JH, Kim SW, Kim SY, Shin CS (2016). Trabecular bone score as a skeletal fragility index in acromegaly patients. Osteoporos Int.

[66] Calatayud M, Pérez-Olivares Martín L, Librizzi MS, Lora Pablos D, González Méndez V, Aramendi Ramos M (2021). Trabecular bone score and bone mineral density in patients with long-term controlled acromegaly. Clin Endocr.

[67] Sorohan MC, Dusceac R, Sorohan BM, Caragheorgheopol A, Poiana C (2021). Trabecular bone score and bone mineral density in acromegalic osteopathy assessment: A cross-sectional study. Arch Osteoporos.

[68] Sala E, Malchiodi E, Carosi G, Verrua E, Cairoli E, Ferrante E (2021). Spine bone texture assessed by trabecular bone score in active and controlled acromegaly: A prospective study. J Endocr Soc.

[69] Donaldson AA, Feldman HA, O’Donnell JM, Gopalakrishnan G, Gordon CM (2015). Spinal bone texture assessed by trabecular bone score in adolescent girls with anorexia nervosa. J Clin Endocrinol Metab.

[70] Modan-Moses D, Megnazi O, Tripto-Shkolnik L, Talmor H, Toledano A, Shilton T (2022). Changes in trabecular bone score and bone density in female adolescents with anorexia nervosa: a longitudinal study. J Clin Densitom.

[71] Batista SL, de Araújo IM, Carvalho AL, Alencar MAVSD, Nahas AK, Elias J (2019). Beyond the metabolic syndrome: Visceral and marrow adipose tissues impair bone quantity and quality in Cushing’s disease. PLoS One.

[72] Stachowska B, Halupczok-Żyła J, Kuliczkowska-Płaksej J, Syrycka J, Bolanowski M (2020). Decreased trabecular bone score in patients with active endogenous Cushing’s syndrome. Front Endocrinol.

[73] Gonzalez Rodriguez EG, Lamy O, Stoll D, Metzger M, Preisig M, Kuehner C (2017). High evening cortisol level is associated with low tbs and increased prevalent vertebral fractures: Osteolaus study. J Clin Endocrinol Metab.

[74] Kim BJ, Kwak MK, Ahn SH, Kim H, Lee SH, Koh JM (2018). Lower trabecular bone score in patients with primary aldosteronism: Human skeletal deterioration by aldosterone excess. J Clin Endocrinol Metab.

[75] Paggiosi MA, Peel NFA, Eastell R (2015). The impact of glucocorticoid therapy on trabecular bone score in older women. Osteoporos Int.

[76] Leib ES, Winzenrieth R (2016). Bone status in glucocorticoid-treated men and women. Osteoporos Int.

[77] Koumakis E, Avouac J, Winzenrieth R, Toth E, Payet J, Kahan A (2015). Trabecular bone score in female patients with systemic sclerosis: Comparison with rheumatoid arthritis and influence of glucocorticoid exposure. J Rheumatol.

[78] Eller-Vainicher C, Morelli V, Ulivieri FM, Palmieri S, Zhukouskaya VV, Cairoli E (2012). Bone quality, as measured by trabecular bone score in patients with adrenal incidentalomas with and without subclinical hypercortisolism. J Bone Miner Res.

[79] Florez H, Hernández-Rodríguez J, Muxi A, Carrasco JL, Prieto-González S, Cid MC (2020). Trabecular bone score improves fracture risk assessment in glucocorticoid-induced osteoporosis. Rheumatology.

[80] Chuang MH, Chuang TL, Koo M, Wang YF (2017). Trabecular bone score reflects trabecular microarchitecture deterioration and fragility fracture in female adult patients receiving glucocorticoid therapy: A pre-post controlled study. BioMed Res Int.

[81] Lee KA, Kim J, Kim HJ, Kim HS (2021). Discriminative ability of trabecular bone score over bone mineral density for vertebral and fragility fracture in patients treated with long-term and low-dose glucocorticoid. Int J Rheum Dis.

[82] Corrado A, Rotondo C, Mele A, Cici D, Maruotti N, Sanpaolo E (2021). Influence of glucocorticoid treatment on trabecular bone score and bone remodeling regulators in early rheumatoid arthritis. Arthritis Res Ther.

[83] Nowakowska-Płaza A, Wroński J, Sudoł-Szopińska I, Głuszko P (2021). Clinical Utility of trabecular bone score (TBS) in fracture risk assessment of patients with rheumatic diseases treated with glucocorticoids. Hormone and metabolic research. Horm Metab.

[84] Gao Y, Wang O, Guan W, Wu X, Mao J, Wang X (2021). Bone mineral density and trabecular bone score in patients with 21-hydroxylase deficiency after glucocorticoid treatment. Clin Endocr.

[85] Bréban S, Briot K, Kolta S, Paternotte S, Ghazi M, Fechtenbaum J (2012). Identification of rheumatoid arthritis patients with vertebral fractures using bone mineral density and trabecular bone score. J Clin Densitom.

[86] Kim D, Cho SK, Kim JY, Choi YY, Sung YK (2016). Association between trabecular bone score and risk factors for fractures in Korean female patients with rheumatoid arthritis. Modern Rheumatology.

[87] Richards C, Leslie WD (2022). Trabecular bone score in rheumatic disease. Curr Rheumatol Rep.

[88] Pedrazzoni M, Casola A, Verzicco I, Abbate B, Vescovini R, Sansoni P (2014). Longitudinal changes of trabecular bone score after estrogen deprivation: Effect of menopause and aromatase inhibition. J Endocrinol Invest.

[89] Naylor KL, Lix LM, Hans D, Garg AX, Rush DN, Hodsman AB (2016). Trabecular bone score in kidney transplant recipients. Osteoporos Int.

[90] Brunerová L, Ronová P, Verešová J, Beranová P, Potoèková J, Kasalický P (2016). Osteoporosis and impaired trabecular bone score in hemodialysis patients. Kidney Blood Press Res.

[91] Yun HJ, Ryoo SR, Kim JE, Choi YJ, Park I, Shin GT (2020). Trabecular bone score may indicate chronic kidney disease-mineral and bone disorder (CKD-MBD) phenotypes in hemodialysis patients: A prospective observational study. BMC Nephrol.

[92] Moon JH, Kim KM, Oh TJ, Choi SH, Lim S, Park YJ (2017). The effect of TSH suppression on vertebral trabecular bone scores in patients with differentiated thyroid carcinoma. J Clin Endocrinol Metab.

[93] Hawkins Carranza F, Guadalix Iglesias S, Luisa De Mingo Domínguez M, Martín-Arriscado Arroba C, López Álvarez B, Allo Miguel G (2020). Trabecular bone deterioration in differentiated thyroid cancer: Impact of long-term TSH suppressive therapy. Cancer Medicine.

[94] Sousa BÉCA, Silva BC, de Oliveira Guidotti T, Pires MC, Soares MMS, Kakehasi AM (2021). Trabecular bone score in women with differentiated thyroid cancer on long-term TSH-suppressive therapy. J Endocrinol Invest.

[95] Baldini M, Ulivieri FM, Forti S, Serafino S, Seghezzi S, Marcon A (2014). Spine bone texture assessed by trabecular bone score (TBS) to evaluate bone health in thalassemia major. Calcif Tissue Int.

[96] Teawtrakul N, Chukanhom S, Charoensri S, Somboonporn C, Pongchaiyakul C (2020). The Trabecular bone score as a predictor for Thalassemia-induced vertebral fractures in northeastern Thailand. Anemia.

[97] McGinty T, Cotter AG, Sabin CA, Macken A, Kavanagh E, Compston J (2019). Assessment of trabecular bone score, an index of bone microarchitecture, in HIV positive and HIV negative persons within the HIV UPBEAT cohort. PLoS One.

[98] Kim YJ, Kang KY, Shin J, Jun Y, Kim SL, Kim YR (2020). Trabecular bone scores in young HIV-infected men: A matched case-control study. BMC Musculoskel Disord.

[99] Tahani N, Nieddu L, Prossomariti G, Spaziani M, Granato S, Carlomagno F (2018). Long-term effect of testosterone replacement therapy on bone in hypogonadal men with Klinefelter Syndrome. Endocrine.

[100] Eller-Vainicher C, Bassotti A, Imeraj A, Cairoli E, Ulivieri FM, Cortini F (2016). Bone involvement in adult patients affected with Ehlers-Danlos syndrome. Osteoporos Int.

[101] Costa R, de Asúa DR, Gullón A, de Miguel R, Bautista A, García C (2021). Volumetric BMD by 3D-DXA and trabecular bone score in adults with Down syndrome. J Clin Densitom.

[102] Alvarenga PPM, Silva BC, Diniz MP, Leite MB, da Silva CAM, de Cássia Mendes Eleutério J (2019). Trabecular bone score: A useful clinical tool for the evaluation of skeletal health in women of short stature. Endocrine.

[103] Jiang N, Xia W (2018). Assessment of bone quality in patients with diabetes mellitus. Osteoporos Int.

[104] Melton LJ, Leibson CL, Achenbach SJ, Therneau TM, Khosla S (2008). Fracture risk in type 2 diabetes: Update of a population-based study. J Bone Miner Res.

[105] Vilaca T, Schini M, Harnan S, Sutton A, Poku E, Allen IE (2020). The risk of hip and non-vertebral fractures in type 1 and type 2 diabetes: A systematic review and meta-analysis update. Bone.

[106] Koromani F, Oei L, Shevroja E, Trajanoska K, Schoufour J, Muka T (2020). Vertebral fractures in individuals with type 2 diabetes: More than skeletal complications alone. Diabetes Care.

[107] Vestergaard P (2007). Discrepancies in bone mineral density and fracture risk in patients with type 1 and type 2 diabetes: A meta-analysis. Osteoporos Int.

[108] Ma L, Oei L, Jiang L, Estrada K, Chen H, Wang Z (2012). Association between bone mineral density and type 2 diabetes mellitus: A meta-analysis of observational studies. Eur J Epidemiol.

[109] Schwartz AV, Vittinghoff E, Bauer DC, Hillier TA, Strotmeyer ES, Ensrud KE (2011). Association of BMD and FRAX score with risk of fracture in older adults with type 2 diabetes. JAMA.

[110] Farr JN, Khosla S (2016). Determinants of bone strength and quality in diabetes mellitus in humans. Bone.

[111] Zhukouskaya V, Ellen-Vainicher C, Gaudio A, Privitera F, Cairoli E, Ulivieri FM (2016). The utility of lumbar spine trabecular bone score and femoral neck bone mineral density for identifying asymptomatic vertebral fractures in well-compensated type 2 diabetic patients. Osteoporos Int.

[112] Bonaccorsi G, Fila E, Messina C, Maietti E, Ulivieri FM, Caudarella R (2017). Comparison of trabecular bone score and hip structural analysis with FRAX® in postmenopausal women with type 2 diabetes mellitus. Aging Clin Exp Res.

[113] Caffarelli C, Giambelluca A, Ghini V, Francolini V, Pitinca MDT, Nuti R (2017). In type-2 diabetes subjects trabecular bone score is better associated with carotid intima-media thickness than BMD. Calcif Tissue Int.

[114] Xue Y, Baker AL, Nader S, Orlander P, Sanchez AJ, Kellam J (2018). Lumbar spine trabecular bone score (TBS) reflects diminished bone quality in patients with diabetes mellitus and oral glucocorticoid therapy. J Clin Densitom.

[115] Holloway KL, de Abreu LLF, Hans D, Kotowicz MA, Sajjad MA, Hyde NK (2018). Trabecular bone score in men and women with impaired fasting glucose and diabetes. Calcif Tissue Int.

[116] Rianon N, Ambrose CG, Buni M, Watt G, Reyes-Ortiz C, Lee M (2018). Trabecular bone score is a valuable addition to bone mineral density for bone quality assessment in older Mexican American women with type 2 diabetes. J Clin Densitom.

[117] Ho-Pham LT, Tran B, Do AT, Nguyen TV (2019). Association between pre-diabetes, type 2 diabetes and trabecular bone score: The Vietnam Osteoporosis study. Diabetes Res Clin Pract.

[118] Neumann T, Lodes S, Kästner B, Lehmann T, Hans D, Lamy O (2016). Trabecular bone score in type 1 diabetes: A cross-sectional study. Osteoporos Int.

[119] Wagh A, Ekbote V, Khadilkar V, Khadilkar A (2021). Trabecular bone score has poor association with pQCT derived trabecular bone density in Indian children with type 1 diabetes and healthy controls. J Clin Densitom.

[120] Mitchell DM, Caksa S, Joseph T, Bouxsein ML, Misra M (2020). Elevated HbA1c is associated with altered cortical and trabecular microarchitecture in girls with type 1 diabetes. J Clin Endocrinol Metab.

[121] Thangavelu T, Silverman E, Akhter MP, Lyden E, Recker RR, Graeff-Armas LA (2020). Trabecular bone score and transilial bone trabecular histomorphometry in type 1 diabetes and healthy controls. Bone.

[122] Choi YJ, Lee HY, Yoon D, Kim A, Shin YS, Park HS (2019). Trabecular bone score is more sensitive to asthma severity and glucocorticoid treatment than bone mineral density in asthmatics. Allergy Asthma Immunol Res.

[123] Kolta S, Briot K, Fechtenbaum J, Paternotte S, Armbrecht G, Felsenberg D (2014). TBS result is not affected by lumbar spine osteoarthritis. Osteoporos Int.

[124] Wildberger L, Boyadzhieva V, Hans D, Stoilov N, Rashkov R, Aubry-Rozier B (2017). Impact of lumbar syndesmophyte on bone health as assessed by bone density (BMD) and bone texture (TBS) in men with axial spondyloarthritis. Joint Bone Spine.

[125] Dufour R, Winzenrieth R, Heraud A, Hans D, Mehsen N (2013). Generation and validation of a normative, age-specific reference curve for lumbar spine trabecular bone score (TBS) in French women. Osteoporos Int.

[126] Hsu Y, Hsieh TJ, Ho CH, Lin CH, Chen CKH (2021). Effect of compression fracture on trabecular bone score at lumbar spine. Osteoporos Int.

[127] White R, Binkley N, Krueger D (2018). Effect of vertebral exclusion on TBS and FRAX calculations. Arch Osteoporos.

[128] Yu EW, Thomas BJ, Brown JK, Finkelstein JS (2012). Simulated increases in body fat and errors in bone mineral density measurements by DXA and QCT. J Bone Miner Res.

[129] Amnuaywattakorn S, Sritara C, Utamakul C, Chamroonrat W, Kositwattanarerk A, Thamnirat K (2016). Simulated increased soft tissue thickness artefactually decreases trabecular bone score: A phantom study Clinical rheumatology and osteoporosis. BMC Musculoskelet Disord.

[130] Cornier MA, Després JP, Davis N, Grossniklaus DA, Klein S, Lamarche B (2011). Assessing adiposity: A scientific statement from the American Heart Association. Circulation.

[131] Shevroja E, Aubry-Rozier B, Hans G, Rodriguez EG, Stoll D, Lamy O (2019). Clinical performance of the updated trabecular bone score (TBS) algorithm, which accounts for the soft tissue thickness: The OsteoLaus study. J Bone Miner Res.

